# A Special Issue

**DOI:** 10.5681/joddd.2010.001

**Published:** 2010-03-14

With the establishment of the online publishing system of Journal of Dental Research, Dental Clinics, Dental Prospects (JODDD), the journal started to enjoy a broader audience outside its publishing home in Iran. In fact, visitors to the JODDD website from other English, and even non-English-speaking countries, outnumber visitors from Iran, which again puts an emphasis on the importance of scholarly communication in an international language, despite the fact that publishing in English by non-natives has been quite an effort for the editorial team. Nonetheless, the editorial team of JODDD is glad to be able to publish a journal that can attract an international readership.

In the third volume of the journal, we published articles from international contributors outside Iran. We are looking forward to seeing more submissions from all around the world, and hope that JODDD will become a forum for researchers, clinicians and educators in the field of dentistry and related sciences in this part of the world. 

We are optimizing our process to help authors prepare manuscripts that can be quickly and efficiently reviewed, edited, and finally published. We have made efforts to reduce the time for reviewing of manuscripts, which will result in the timely publication of research results.

In addition to the Expert Medica database for health professionals (EMCARE), JODDD is now indexed in WHO Index Medicus for the Eastern Mediterranean Region (IMEMR), the Directory of Open Access Journals (DOAJ) and Index Copernicus. With higher visibility that these databases bring to the articles published in JODDD in addition to the Google Scholar search engine, authors will enjoy a broader readership for their articles.

In this issue, we have focused on a pre-malignant lesion that often degrades the quality of life of the involved individuals. Oral lichen planus is important for the dental practitioner from multiple standpoints. Lichen planus is a chronic inflammatory and immunologic mucocutaneous lesion. Patients usually complain of lichen planus symptoms following the onset of the symptoms and signs for the rest of their lives. It is considered a pre-malignant lesion, and therefore, its diagnosis and treatment need special attention. Tabriz University of Medical Sciences, which is home to JODDD, has been planning to be recognized as the national reference center for the lichen planus.

We are grateful to Dr. Ardeshir Lafzi, the Editor-in-Chief, who is leaving Tabriz University of Medical Sciences, and this oral lichen planus special issue is the last issue edited by Dr. Lafzi. The fourth volume of the journal will continue under the editorship of Dr. Naser Asl Aminabadi, associate professor, Department of Pediatric Dentistry, Tabriz University of Medical Sciences. We also have to thank the members of our editorial board, who have fulfilled their three-year assignment, and our reviewers, who are listed in the appendix of this issue. We hope to have more international editorial board members, the presence of whom will encourage other professionals in the field to participate in shaping our journal and cooperation for the advancement of sharing knowledge in this field.

